# Morphological and Functional Aspects and Quality of Life in Patients with Achromatopsia

**DOI:** 10.3390/jpm13071106

**Published:** 2023-07-07

**Authors:** Caroline Chan, Berthold Seitz, Barbara Käsmann-Kellner

**Affiliations:** Department of Ophthalmology, University of Saarland Medical Center in Homburg/Saar, 66421 Homburg/Saar, Germany; caroline.chan@uks.eu (C.C.); berthold.seitz@uks.eu (B.S.)

**Keywords:** achromatopsia, cone, low vision aids, quality of life, personalised ophthalmology

## Abstract

(1) Background: Achromatopsia is a rare disease of which the natural course and impact on life are still unknown to this date. We aimed to assess the morphological, functional characteristics, and quality of life in a large sample size of patients with achromatopsia. (2) A total of 94 achromats were included in this retrospective cohort study. Sixty-four were patients of the Department of Ophthalmology, Saarland University Medical Centre in Homburg/Saar, Germany, between 2008 and 2021. Thirty further participants with achromatopsia from the national support group were included using an online questionnaire, which is available under ‘Supplementary data’. Statistical analysis was performed using SPSS Version 25; (3) The 94 patients (37 males (39.4%) and 57 females (60.6%)) showed a mean age of 24.23 ± 18.53 years. Visual acuity was stable (SD ± 0.22 logMAR at 1.0 logMAR) over a time of observation from 2008 to 2021. Edge filter glasses were the most used optical aids, while enlarged reading glasses were the most used low vision aids. (4) Conclusions: Our findings give an insight into describing the natural process and the quality of life of achromatopsia. The results demonstrate that achromatopsia is a predominantly stationary disease. The individual prescription of edge filters and low-vision aids is essential following a personalised fitting.

## 1. Introduction

Our study’s scope was to analyse the morphological changes that may be associated with achromatopsia, to follow these changes in visual acuity, and to assess the impact of achromatopsia on patients’ quality of life. As far as we know, to this date, the nature of achromatopsia has still not been clearly defined. Furthermore, no study on achromatopsia has been conducted with a large sample size focusing on the psychosocial aspect. Achromatopsia, also called rod monochromatism or total colour blindness, belongs to cone dysfunction syndromes and is present at birth [1]. It is considered a rare disease with a prevalence of 1/30,000 births worldwide [2]. It is an inherited, autosomal recessive disease, with typical characteristics such as a congenital reduction in visual acuity to around 20/200, photophobia, lack of colour discrimination, and nystagmus. The fundus examination usually does not show pathological changes, but sometimes there can be a constriction of blood vessels and pathological macular findings in the adult age [3,4,5]. Mutations of the six genes *CNGA3* [6], *CNGB3* [7], *GNAT2* [8], *PDE6H* [9], *PDE6C* [10], which play a vital role in the phototransduction cascade and *ATF6* [11], which regulates the unfolded protein response, have been identified in achromatopsia. 

Refractive errors are common [12]. There are two types of achromatopsia: In ‘complete’ achromatopsia, all three types of cones (1. Blue, 2. Green, 3. Red) do not function, while in ‘incomplete’ achromatopsia, there may be the partial function of one or more cone types [13].

**Figure 1:**
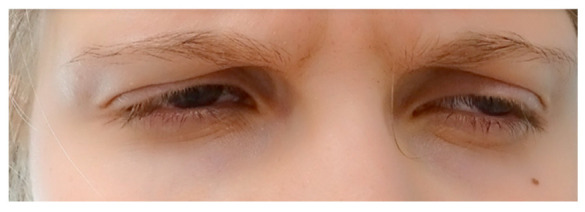
A 15-year-old girl without edge filter, not making eye contact.

**Figure 2:**
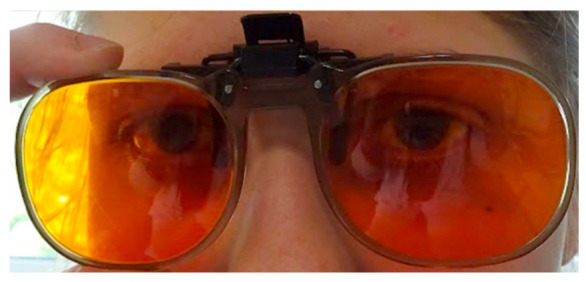
Same girl with edge filter glasses, eyes opened normally and eye contact [16].

Up to this date, achromatopsia treatment has mainly focused on managing the symptoms with different optical and electronic low-vision aids [3,14]. The first genetic treatment studies for *CNGA3* and *CNGB3* are underway; however, the results in adults have not shown vigorous improvements. While there is currently no known cure, many management aids can alleviate some symptoms associated with ACHM. In particular, photoaversion has been described to be one of the more debilitating symptoms of achromatopsia, with the need for different aids [15]. (Figure 1) These include red-tinted contact lenses, sunglasses, wrap-around glasses, and dark and red-filtered glasses, which have been shown to reduce photophobia and improve visual acuity. These optical aids have to be tried and fitted individually for the best benefit of the patient. Especially red-tinted contact lenses have been a relief for achromats, as they are more discrete than dark and red-filtered glasses. (Figure 2) Low vision aids such as high-powered magnifiers and electronic devices can be useful for reading. Children with ACHM should be given preferential seating in class to benefit fully from these aids (such as at the front and to avoid the impact of glare on vision or away from windows) [2,14].

It has recently been shown that gene therapy using an adeno-associated virus could perhaps provide a promising treatment for achromatopsia. Recently, human clinical trials have been developed, which primarily showed the safety and efficacy of the treatment [17,18,19]. There is still an ongoing debate concerning the natural course of achromatopsia. Some studies report that it is a progressive disorder, suggesting that gene therapy should be applied at an earlier age [20,21,22,23,24]. By contrast, other studies imply that it is a predominantly stationary condition, indicating that there might be a larger window for gene therapy [25,26,27,28]. However, these studies had small group sizes, with Hirji et al. having the largest with 50 subjects [28]. Furthermore, research has rarely been performed on the quality of life and subjective impressions of patients with achromatopsia. Very few studies have evaluated the influence of complete colour vision loss on the quality of life of these people [29]. The only study that came close to quality of life was Aboshiha et al.’s, which investigated the impact of photophobia on the life of achromats [15]. With human gene therapy trials developing rapidly, it is fundamental to elucidate the natural process of achromatopsia and find correlations between the genotype and phenotype to choose suitable candidates and the right time for treatment. In addition, understanding how achromatopsia affects the lives of these patients could help in identifying strategies that can improve patient outcomes.

## 2. Materials and Methods

### 2.1. Participants

In this retrospective cohort study, a total of 94 participants with a confirmed diagnosis of achromatopsia were included. The diagnosis was established by clinical presentation (severe glare sensitivity with normal ocular findings, in several cases with electrophysiological confirmation and with genetic evaluation). Exclusion criteria included atypical clinical findings (no glare sensitivity), no acceptance of edge filters, morphologically visible pathological findings (such as in albinism which presents with glare sensitivity as well), and negative genetic testing in patients who were genetically tested. Sixty-four of the participants were patients of the Department of Ophthalmology, Saarland University Medical Centre in Homburg/Saar, Germany, between 2008 and 2021. Thirty additional participants who completed a standardized questionnaire were members of the national support association of achromatopsia. The age range was very broad as we aimed to include all patients with this rare disease. The research was approved by the local ethics committee of Saarland (Nr. 85-21) and followed the principles of the Declaration of Helsinki. Informed consent was provided by all subjects.

### 2.2. Medical Records

Data from the patient’s medical history were collected using the clinic’s electronic charts. Patients underwent a complete ophthalmic examination: Visual acuity (Landolt eye chart), colour vision (Ishihara and Panel D 15 test), refraction (retinoscopy), anterior segment findings (slit-lamp), central and peripheral retina and optic nerve head (ophthalmoscope), and strabismus (prism cover test).

### 2.3. Questionnaire

A survey was conducted from April to May 2021 to analyse the quality of life that people living with achromatopsia had. This survey was distributed to all of the patients in our hospital diagnosed with achromatopsia and to the members of the national self-help group of achromatopsia (Achromatopsie Selbsthilfe e.V. Dorsten, Germany, www.achromatopsie.de, accessed on 20 March 2021). This survey consisted of 18 main questions comprising demographic and non-demographic questions based on their quality of life, such as education and visual aids (Table 1).

Most demographic questions were multiple-choice with a few open-ended questions. A free-text box was added after each question to let participants share any additional thoughts or information. The following criteria were retrieved from their medical history, and the questionnaire was taken into consideration: gender, age, age of diagnosis, past visual acuity, actual visual acuity, standardised colour test, subjective colour perception, refraction, morphological findings, strabismus, academic career, practiced occupation, present low vision aids, degree of handicap, allowances for the blind, mutated gene and primary concern of the patients.

### 2.4. Statistical Analysis

The software SPSS Statistics, IBM, Version 25, was used to enter and evaluate our data. Continuous data were described as the mean, standard deviation, median, minimum, and maximum. Categorical variables were described as percentages and were compared using the Chi-Square test. The Wilcoxon test was used for non-parametric variables. Spearman tests determined the relationship between non-parametric variables. Spearman’s 𝜌 (rho) is a rank correlation coefficient that was used for two ordinal variables or when one variable had a continuous normal distribution; the other variable was categorical or non-normally distributed. This test was a non-parametric test, which could be used with variables that had a non-normal distribution. *p* values < 0.05 were considered statistically significant.

## 3. Results

We took two approaches to collect the data: patients’ records collected from our clinic’s database and the questionnaire we created for the purpose of this study. These clinical records were used to evaluate morphological and functional characteristics, while the questionnaire assessed the functional aspects and the quality of life of achromats. Out of the 48 clinical examinations performed in domo, three of the patients did not request a genetic analysis (Table 2).

### 3.1. Demographics and Genes

Ninety-four participants were included in this study, with 37 males (39,4%) and 57 females (60.6%). The mean age was 24.23 years (SD ± 18.53 years). In total, 32 (34%) subjects presented with mutations in the *CNGB3* gene, 12 (12.8%) subjects had a *CNGA3* mutation, one (1.1%) subject had the *PDE6C* gene, and for 49 subjects (52.1%), no genetic analysis had been made up to now, mostly because the patients saw no necessity in spite of being informed about ongoing clinical trials.

### 3.2. Functional Findings

To confirm that achromatopsia is a stationary disease (functional aspect), we analysed the changes in visual acuity, as well as the refractive/orthoptic changes, with the increase in age in the achromats.

### 3.3. Visual Acuity

Out of the 62 patients examined ophthalmologically, the first known average visual acuity was 1.0 logMAR (SD ± 0.22), ranging between 0.42 logMAR and 1.52 logMAR. Out of 91 patients, including those answering the questionnaire, the actual average visual acuity was also 1.0 logMAR (SD ± 0.23), ranging from 0.22 logMAR to 1.70 logMAR.

We evaluated the difference between the visual acuity during the first and final visit to our clinic with an observation time of 1 to 21 years. The results indicated no significant difference between both measurements (r = 0.239, *p* = 0.811).

**Figure 3:**
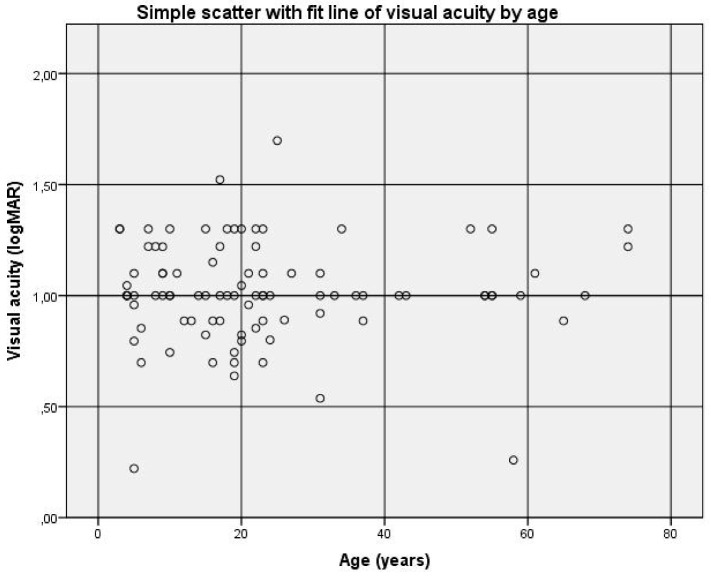
There was no significant decrease in visual acuity as a function of age. Visual acuity is expressed in logMAR and age in years. No correlation was found between visual acuity and age (r = 0.002 and *p* = 0.985).

Out of 91 patients, no correlation was found between visual acuity and age (r = 0.002 and *p* = 0.985) (Figure 3). This confirmed the assumption that achromatopsia is a functionally stable disease over the years.

Out of the 62 patients seen and examined ophthalmologically, 12 had the *CNGB3* gene, 32 had the *CNGB2* gene, 1 had the *PDE6C* gene, and 17 did not have a genetic analysis. We analysed if any of the six genes responsible for achromatopsia were linked to visual impairment. Out of 45 patients who received a genetic diagnosis, no correlation was found between the type of gene and visual acuity (r = −0.158, *p* = 0.311).

### 3.4. Refractive and Orthoptic Findings (Refraction, Nystagmus, Strabismus)

Out of 62 patients, the spherical refraction mean was 1.83 diopters (SD ± 3.76, −4.38 to +8.5 dpt). No correlation was found between the mutated genes and the degree of spherical refraction (r = −0.075, 0.669). The mean astigmatic refraction was −1.86 diopters (SD ± 0.88, −3.0 to −0.5 dpt). No correlation between the visual acuity and spherical refraction was found (r = −0.111, *p* = 0.393), between the visual acuity and astigmatic refraction (r = 0.144, *p* = 0.380), and between the mutated genes and the astigmatic refraction (r = 0.097, *p* = 0.578). Out of 61 patients, 54 (88.5%) had nystagmus, while 7 (11.5%) did not present with nystagmus. No statistical tests were used to analyse the frequency of nystagmus. As many conatal visually handicapped people were present with strabismus, we tested these for achromatopsia as well. Out of 58 patients, 34 (58.6%) did not present any strabismus, 6 (10.3%) presented a strabismus convergens, 17 (29.3%) had a strabismus divergens, and 1 (1.7%) presented a strabismus convergens and verticalis. No correlation was found between age and strabismus (r = 0.057, *p* = 0.672). No correlation was found between the mutated gene and strabismus (r = −0.230, *p* = 0.213).

### 3.5. Morphological Findings

**Figure 4:**
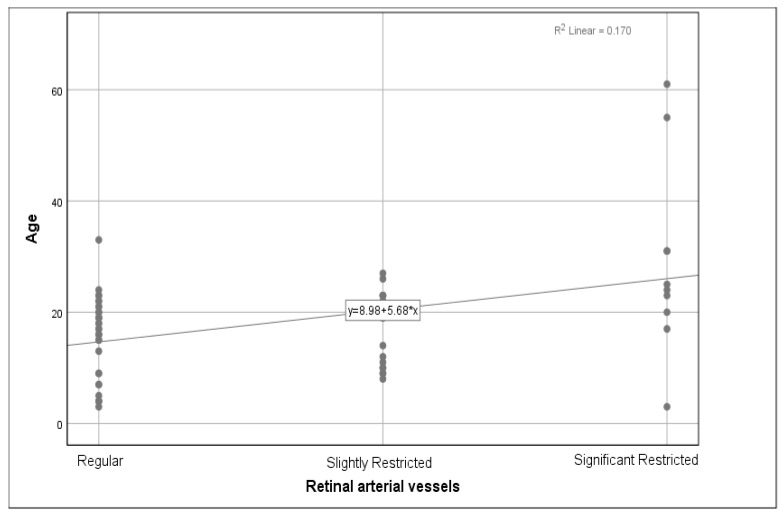
Progressive changes in the retinal arterial vessels as a function of age. A weak relationship between the age and the retinal arterial vessels was found (r = 0.345, *p* = 0.007). No correlation was found between the mutated gene and the retinal vessels (r = −0.157, *p* = 0.375).

We analysed the relationship between the genotype and phenotype, as well as the age and phenotype. The phenotype consisted of changes in the anterior segments, cataracts, retinal arterial vessels, the optic nerve, the peripheral retina, the fundus periphery, and the macula. Out of 62 subjects, a majority of 59 (95.2%) patients did not have any pathological changes in the anterior segment findings, with 3 (4.8%) over 50 years old presenting with a cataract, which was interpreted as an age-related finding and was not associated with achromatopsia. As the three patients with an incipient cataract had no significantly different visual acuity, we did not exclude them from the patient group. Out of 60 subjects, 32 (53.3%) had regular retinal arterial vessels, 18 (30%) had slightly constricted blood vessels, and 10 (16.7%) presented with significant constriction. A weak correlation between age and retinal artery constriction was found (r = 0.345, *p* = 0.007) (Figure 4).

We noted a weak correlation between the age and the optic nerve head morphology (r = 0.280, *p* = 0.027).

Out of 61 patients, the majority of 52 (85.2%) had no abnormal findings in the peripheral retina, while 9 (14.8%) presented deposits around the pigment epithelium and other changes in the fundus periphery. No correlation was found between age and the fundus periphery (r = 0.057, *p* = 0.660) nor between the mutated gene and the fundus periphery (r = −0.135, *p* = 0.438).

For a sample of 62 patients, 23 (37.1%) did not show changes in the macula, and 20 (32.3%) had a well-delimited wall reflex but not a well-delimited foveal reflex. For 16 (25.8%), the macula was not differentiated, and for the remaining 3 (4.8%), the macula was not differentiated with pigment epithelium alterations. We noted no correlation between age and changes in the macula (r = 0.121, *p* = 0.348) and no correlation between the mutated gene and the macula (r = −0.167, *p* = 0.338).

The majority of the 62 patients (*n* = 40, 64.5%) did not present with any pathology of the optic nerve head, while 7 (11.3%) presented with a slightly pale optic nerve head, 3 (4.8%) presented with a noticeably pale optic nerve head, 7 (11.3%) had pronounced retinal optic atrophy and 5 presented nerve head drusen (8.1%). We noted a weak correlation between age and the optic nerve head (r = 0.280, *p* = 0.027) but noted no correlation between the mutated gene and the optic nerve head (r = −0.249, *p* = 0.148).

### 3.6. Quality of Life

#### 3.6.1. Handicap and Allowances

Achromats are assigned a visual handicap ranging from 0% to 100% according to German regulations for visually impaired citizens. Usually, the amount of allowance received depends on the degree of the handicap. Out of 61 patients, 17 (27.9%) had a 100% degree of visual handicap, 10 (16.4%) had a 90% degree of visual handicap, 14 (23%) had an 80% degree of visual handicap, 9 (14.8%) had a 70% degree of visual handicap, 1 (1.6%) had a 50% degree of visual handicap and 10 (16.4%) had no degree of visual handicap. This confirmed our clinical impression that the degree of visual handicap could be judged very differently legally in spite of a comparable visual handicap. There was no significant relationship between the degree of handicap as a function of age (*n* = 61, r = 0.140, *p* = 0.280). Out of 67 patients, 10 (14.9%) received allowances for the blind, while 57 (85.1%) did not receive any.

#### 3.6.2. Mean Age of Diagnosis, School Career and Academic Achievements

We ranked academic achievements based on the educational benchmarks completed, such as a high school diploma or university degree. We analysed the relationship between the academic achievements of achromats with the age of diagnosis and their attendance at mainstream schools or for those visually handicapped students. The mean age when the diagnosis of achromatopsia was established was 6.49 years (SD ± 1.50), with the oldest patient, age 74, diagnosed at the age of 71 and the youngest patient having been diagnosed shortly after birth. Out of 56 respondents, the majority went to a mainstream school (*n* = 40; 71.4%), while fewer went to schools for the visually impaired (*n* = 9; 16.1%) or a mix of specialised and mainstream schools (*n* = 7; 12.5%).

We received data on the academic qualifications from 28 respondents and noted that 26 had a high school diploma (92.9%), 1 (3.6%) did not have a school qualification, and 1 (3.6%) obtained a middle school diploma. Regarding further education, we received 26 responses, which consisted of one (3.8%) having an apprenticeship, another (3.8%) doing higher intermediate service, and the majority pursuing university studies (*n* = 24; 92.3%). The participants had different job occupations, most working in social professions. No correlation was found between the age of diagnosis established with a school qualification (r = 0.273, *p* = 0.168) or further education (r = −0.335, *p* = 0.102). We did not find any correlation between the type of school and the kind of school qualification achieved (r = −0.124, *p* = 0.530) and between the type of school and further education (r = −0.206, *p* = 0.313).

#### 3.6.3. Use of Optical Aids and Low Vision Aids

For present optical and low vision aids, out of 87 subjects, 27 (31%) had optical aids, 55 (63.2%) used a combination of optical and low vision aids, and 5 (5.7%) had none. Many patients used several types of edge filter glasses following individual customisation and fitting (Table 3 and Table 4).

#### 3.6.4. Primary Impairing Issue of Achromatopsia (Low Vision, Glare Sensitivity, No Colour Vision) of Patients with Achromatopsia

For the question ‘What is your primary concern?’, out of 43 respondents, the primary wish of 26 (60.5%) achromats was normal visual acuity. In total, 15 (34.9%) achromats wished for no more glare sensitivity, and only 2 (4.7%) wanted intact colour vision.

This was corroborated by the response from 44 respondents concerning the use of a colour system: 30 did not use any, 7 used colorADD, and 7 used a different colour system.

## 4. Discussion

Our findings suggest that age does not influence visual acuity and that visual acuity usually does not change with age (mean = 1.0 logMAR). These findings are in accordance with most prior studies [20,22,23,25,28,30], which have also concluded that visual acuity becomes stable over time but differs from a few other studies that concluded a decline in visual acuity [21,24]. Refractive error and nystagmus were common traits in our patients, with hyperopia being the most common refractive error. The data we collected indicated no correlation between the degree of spherical refraction and astigmatism against visual acuity.

Regarding ocular morphology, we observed a subtle constriction of the retinal arterial vessels with age, which could be a feature found in achromatopsia [3]. A constriction of the qualitative calibre of the retinal vasculature could, however, also be due to age. The optic nerve also presented morphological changes with age, suggesting retinal atrophy. We found no relationship between fundus periphery changes with age and between macula changes and age. These findings suggest that although progressive retinal changes could be seen, they are likely to develop slowly. This correlates to prior studies [25,26,27,28], which observed minor macular changes over time and demonstrated that achromatopsia is a predominantly stationary disorder.

The majority of patients with available genetic data had either a change in the *CNGA3* or the *CNGB3* gene. This finding corresponds to a past study on achromatopsia in which these genes accounted for more than 70% of cases [31]. We did not observe any phenotype–genotype relationship, indicating that the clinical characteristics for genetic mutations of *CNGA3* and *CNGB3* were similar, which is in accordance with past studies [20,21,24,26,27,28].

With the mean age of diagnosis being established at 6.49 years old, we can say that although the symptoms of achromatopsia were present at birth or early infancy [1], it is often diagnosed later in childhood. Most of the participants were diagnosed by specialised eye departments. Two of our patients even self-diagnosed (internet research) and were later confirmed after consulting an eye doctor who did not identify the disease, indicating that the disease is still not well known among non-specialised ophthalmologists. However, we noted that the diagnosis was found at a later age in older patients. We can, therefore, say that achromatopsia has only recently become better known and is now diagnosed much earlier than in the past. Most of the participants went to regular school, obtained a high school diploma, and attended university, which shows that achromats are not academically hindered. We also noted that attending schools for the visually impaired is not correlated with better or worse academic achievement in this patient population.

Additionally, an earlier age of diagnosis did not influence education, confirming that achromats are not academically handicapped. However, it would be misleading to conclude that achromatopsia is not a handicap in society. There might be a limitation in our study as there could be a greater tendency to answer the questionnaire by academically more proficient people.

The fact that the degree of handicap varied among our patients with a very uniform visual acuity shows that the given degree of handicap is often inappropriate. It should be a goal in the legal classification of (any) handicap to treat and classify patients only following standardised tests and personality-independent examinations. Many participants have indicated that they have problems obtaining aids or allowances for the blind because institutions only consider visual acuity for the degree of handicap in achromats and not colour vision deficits and debilitating photophobia.

According to our results, most patients with achromatopsia need to use optical aids for their symptoms, highlighting the importance of an early and correct diagnosis to manage the symptoms with the appropriate aids. These visual aids have to be fitted in a personalised matter to alleviate the individual symptoms of each patient. Edge filter glasses are the most used, with contact lenses being less used among our patients. This could be explained by the fact that edge filters combined with contact lenses are not accepted by German health insurance regulations and can, therefore, be quite expensive. Additionally, many of the participants indicated in the survey that they would try to avoid edge filter glasses outdoors to avoid social discrimination, showing that achromats try to adapt to society. Hence, contact lenses might be a practical option but costly. Reading glasses and a simple magnifying glass are the most used low-vision aids as they are easier to use than electronic devices. Many patients use these new technologies (smartphones, tablets) as low-vision aids.

Normal visual acuity is the first wish for most patients, followed by a reduction in glare sensitivity and colour vision. These findings are in accordance with Aboshiha et al. [15], who also found out that good visual acuity was the main wish for most achromats, followed by glare sensitivity and colour vision. Furthermore, most participants did not use a colour recognition system. These findings indicate that colour is not the most debilitating symptom in total colour blindness but visual acuity and glare sensitivity are contrary to what one might expect.

In summary, our study suggests that achromatopsia is a relatively stable disease with only subtle morphological changes. Concerning the quality of life, people need to be sensitised, as the handicap arising from achromatopsia is still ambiguous. To our knowledge, this study has the largest sample size for this rare disease with a wide range of ages. Thus, we could make critical statistical comparisons within the group. It was also the first to assess the overall quality of life of patients living with achromatopsia. The limitation was the retrospective aspect with missing information in medical charts, non-respondents to the survey, and subjective answers, which could lead to missing key data and subjective bias.

A personalised approach in achromatopsia is essential when evaluating the need for individual low vision aids and in testing the kind of edge filter. Only this personalised approach can ensure the best development for children and the best working conditions for adults.

In our study, the natural history of achromatopsia is based only on a limited 13-year period in most participants of a younger age. Future longitudinal studies with an extended follow-up period and the inclusion of older patients are needed to strengthen the claim that achromatopsia is a stationary disease. Furthermore, studies with a large sample size are needed to confirm our findings and further analyse individual visual aids and achromats’ quality of life.

## Figures and Tables

**Table 1:** The 18 main questions asked in our questionnaire to individuals with achromatopsia.

(1). Are you a member in a visually impaired association?
(2). What is your age and gender?
(3). Where was your ophthalmologic treatment?
(4). Was your diagnosis of achromatopsia genetically confirmed?
(5). When was the diagnosis of achromatopsia made?
(6). What is your visual acuity?
(7). What is/was your school education?
(8). What is/was your vocational training?
(9). What is/was your profession?
(10). Do you own a disability ID card?
(11). Do you receive any blind allowances?
(12). Which optical aids/light protection do you use?
(13). Which magnifying aids do you use?
(14). Which other aids do you use?
(15). Which computer programs/apps do you use?
(16). Do you use a colour naming system?
(17). What is the visual issue you suffer most from?
(18). How is your personal experience living with achromatopsia?

**Table 2:** Method of data collection for the population of participants.

	Total Number out of 94 Achromats	Percentage Number out of 94 Achromats
Only clinical examinations performed in domo	48	51%
Out of clinical examinations performed in domo; Molecular genetics confirmed	45	48%
Clinical examinations performed in domo + answered questionnaire	16	17%
Only answered questionnaire	30	32%

**Table 3:** Relative proportions of optical aids used *n* = 87 patients.

	Number out of 87 Patients	% out of 87 Patients
Optical aids (for refractive error and glare sensitivity)		
Edge filter glasses with diopter correction	38	43.7%
Edge filter glasses without diopter correction	37	42.5%
Tinted glasses without edge filters, usually sunglasses	22	25.3%
Edge filter contact lenses with diopter correction	13	14.9%
Side Protection	10	11.5%
Contact lenses with diopter values for optical correction	8	9.2%
Edge filter contact lenses without diopter correction	8	9.2%
Overspecs	2	2.3%

**Table 4:** Relative proportions of low vision aids used *n* = 87 patients.

Low Vision Aids		
Reading glasses	49	56.3%
Simple magnifying glass	48	55.2%
Smartphone	34	39.1%
Tablet	33	37.9%
Monocular	32	36.8%
CCTV (close circuit TV)	27	31%
PC	15	17.2%
Magnifying app	13	14.9%
Zoom-Text	13	14.9%
Screen magnifier	12	13.8%
Electronic magnifying glass	10	11.5%

## Data Availability

The data that support the findings of this study are available from the corresponding author upon reasonable request.

## References

[1] Aboshiha J., Dubis A.M., Carroll J., Hardcastle A.J., Michaelides M. (2015). The cone dysfunction syndromes. Br. J. Ophthalmol..

[2] Remmer M.H., Rastogi N., Ranka M.P., Ceisler E.J. (2015). Achromatopsia: A review. Curr. Opin..

[3] Achromatopsia. GeneReviews^®^. https://www.ncbi.nlm.nih.gov/books/NBK1418/.

[4] Tsang S.H., Sharma T. (2018). Rod Monochromatism (Achromatopsia). Adv. Exp. Med. Biol..

[5] Thomas M.G., Papageorgiou E., Kuht H.J., Gottlob I. (2022). Normal and abnormal foveal development. Br. J. Ophthalmol..

[6] Solaki M., Baumann B., Reuter P., Andreasson S., Audo I., Ayuso C., Balousha G., Benedicenti F., Birch D., Bitoun P. (2022). Comprehensive variant spectrum of the CNGA3 gene in patients affected by achromatopsia. Hum. Mutat..

[7] Weisschuh N., Sturm M., Baumann B., Audo I., Ayuso C., Bocquet B., Branham K., Brooks B.P., Catalá-Mora J., Giorda R. (2020). Deep-intronic variants in CNGB3 cause achromatopsia by pseudoexon activation. Hum. Mutat..

[8] Felden J., Baumann B., Ali M., Audo I., Ayuso C., Bocquet B., Casteels I., Garcia-Sandoval B., Jacobson S.G., Jurklies B. (2019). Mutation spectrum and clinical investigation of achromatopsia patients with mutations in the GNAT2 gene. Hum. Mutat..

[9] Andersen M.K.G., Bertelsen M., Grønskov K., Kohl S., Kessel L. (2023). Genetic and Clinical Characterization of Danish Achromatopsia Patients. Genes.

[10] Weisschuh N., Stingl K., Audo I., Biskup S., Bocquet B., Branham K., Burstedt M.S., De Baere E., De Vries M.J., Golovleva I. (2018). Mutations in the gene PDE6C encoding the catalytic subunit of the cone photoreceptor phosphodiesterase in patients with achromatopsia. Hum. Mutat..

[11] Kohl S., Zobor D., Chiang W.C., Weisschuh N., Staller J., Menendez I.G., Chang S., Beck S.C., Garrido M.G., Sothilingam V. (2015). Mutations in the unfolded protein response regulator ATF6 cause the cone dysfunction disorder achromatopsia. Nat. Genet..

[12] Poloschek C., Kohl S. (2010). Achromatopsie. Ophthalmologe.

[13] Michaelides M., Hunt D., Moore A. (2004). The cone dysfunction syndromes. Br. J. Ophthalmol..

[14] Schornack M., Brown W., Siemsen D. (2007). The use of tinted contact lenses in the management of achromatopsia. Optometry.

[15] Aboshiha J., Kumaran N., Kalitzeos A., Hogg C., Rubin G., Michaelides M. (2017). A Quantitative and Qualitative Exploration of Photoaversion in Achromatopsia. Investig. Ophthalmol. Vis. Sci..

[16] Käsmann-Kellner B., Hoffmann M.B. (2023). Achromatopsia—Clinic, diagnostics, genes, brain and quality of life. Die Ophthalmol..

[17] Fischer M.D., Michalakis S., Wilhelm B., Zobor D., Muehlfriedel R., Kohl S., Weisschuh N., Ochakovski G.A., Klein R., Schoen C. (2020). Safety and Vision Outcomes of Subretinal Gene Therapy Targeting Cone Photoreceptors in Achromatopsia. JAMA Ophthalmol..

[18] Reichel F.F., Michalakis S., Wilhelm B., Zobor D., Muehlfriedel R., Kohl S., Weisschuh N., Sothilingam V., Kuehlewein L., Kahle N. (2021). Three-year results of phase I retinal gene therapy trial for CNGA3-mutated achromatopsia: Results of a non randomised controlled trial. Br. J. Ophthalmol..

[19] Mendell J.R., Al-Zaidy S.A., Rodino-Klapac L.R., Goodspeed K., Gray S.J., Kay C.N., Boye S.L., Boye S.E., George L.A., Salabarria S. (2021). Current Clinical Applications of In Vivo Gene Therapy with AAVs. Mol. Ther..

[20] Thiadens A.A., Somervuo V., van den Born L.I., Roosing S., van Schooneveld M.J., Kuijpers R.W., van Moll-Ramirez N., Cremers F.P., Hoyng C.B., Klaver C.C. (2010). Progressive Loss of Cones in Achromatopsia: An Imaging Study Using Spectral-Domain Optical Coherence Tomography. Investig. Ophthalmol. Vis. Sci..

[21] Thiadens A.A., Slingerland N.W., Roosing S., van Schooneveld M.J., van Lith-Verhoeven J.J., van Moll-Ramirez N., van den Born L.I., Hoyng C.B., Cremers F.P., Klaver C.C. (2009). Genetic Etiology and Clinical Consequences of Complete and Incomplete Achromatopsia. Ophthalmology.

[22] Thomas M.G., Kumar A., Kohl S., Proudlock F.A., Gottlob I. (2011). High-Resolution In Vivo Imaging in Achromatopsia. Ophthalmology.

[23] Thomas M.G., McLean R.J., Kohl S., Sheth V., Gottlob I. (2012). Early signs of longitudinal progressive cone photoreceptor degeneration in achromatopsia. Br. J. Ophthalmol..

[24] Brunetti-Pierri R., Karali M., Melillo P., Di Iorio V., De Benedictis A., Iaccarino G., Testa F., Banfi S., Simonelli F. (2021). Clinical and Molecular Characterization of Achromatopsia Patients: A Longitudinal Study. Int. J. Mol. Sci..

[25] Sundaram V., Wilde C., Aboshiha J., Cowing J., Han C., Langlo C.S., Chana R., Davidson A.E., Sergouniotis P.I., Bainbridge J.W. (2014). Retinal Structure and Function in Achromatopsia. Ophthalmology.

[26] Genead M.A., Fishman G.A., Rha J., Dubis A.M., Bonci D.M.O., Dubra A., Stone E.M., Neitz M., Carroll J. (2011). Photoreceptor Structure and Function in Patients with Congenital Achromatopsia. Investig. Ophthalmol. Vis. Sci..

[27] Aboshiha J., Dubis A.M., Cowing J., Fahy R.T., Sundaram V., Bainbridge J.W., Ali R.R., Dubra A., Nardini M., Webster A.R. (2014). A Prospective Longitudinal Study of Retinal Structure and Function in Achromatopsia. Investig. Ophthalmol. Vis. Sci..

[28] Hirji N., Georgiou M., Kalitzeos A., Bainbridge J.W., Kumaran N., Aboshiha J., Carroll J., Michaelides M. (2018). Longitudinal Assessment of Retinal Structure in Achromatopsia Patients with Long-Term Follow-up. Investig. Ophthalmol. Vis. Sci..

[29] Stoianov M., de Oliveira M.S., dos Santos Ribeiro Silva M.C.L., Ferreira M.H., de Oliveira Marques I., Gualtieri M. (2019). The impacts of abnormal color vision on people’s life: An integrative review. Qual. Life Res..

[30] Georgiou M., Singh N., Kane T., Zaman S., Hirji N., Aboshiha J., Kumaran N., Kalitzeos A., Carroll J., Weleber R.G. (2020). Long-Term Investigation of Retinal Function in Patients with Achromatopsia. Investig. Ophthalmol. Vis. Sci..

[31] Michalakis S., Gerhardt M., Rudolph G., Priglinger S., Priglinger C. (2022). Achromatopsia: Genetics and Gene Therapy. Mol. Diagn. Ther..

